# Supplementary material for: Association between oral microbiome and seven types of cancers in East Asian population: a two-sample Mendelian randomization analysis
Source: Front Mol Biosci. 2023 Nov 21;10:1327893. doi: 10.3389/fmolb.2023.1327893 (PMC10702768; doi:10.3389/fmolb.2023.1327893)
Supplement: Supplementary file 1 [file DataSheet1.ZIP › Supplementary Materials/MR plots for tongue/tongue═╝/Prostate cancer/pheno.1249_to_prostate cancer_forest.pdf]

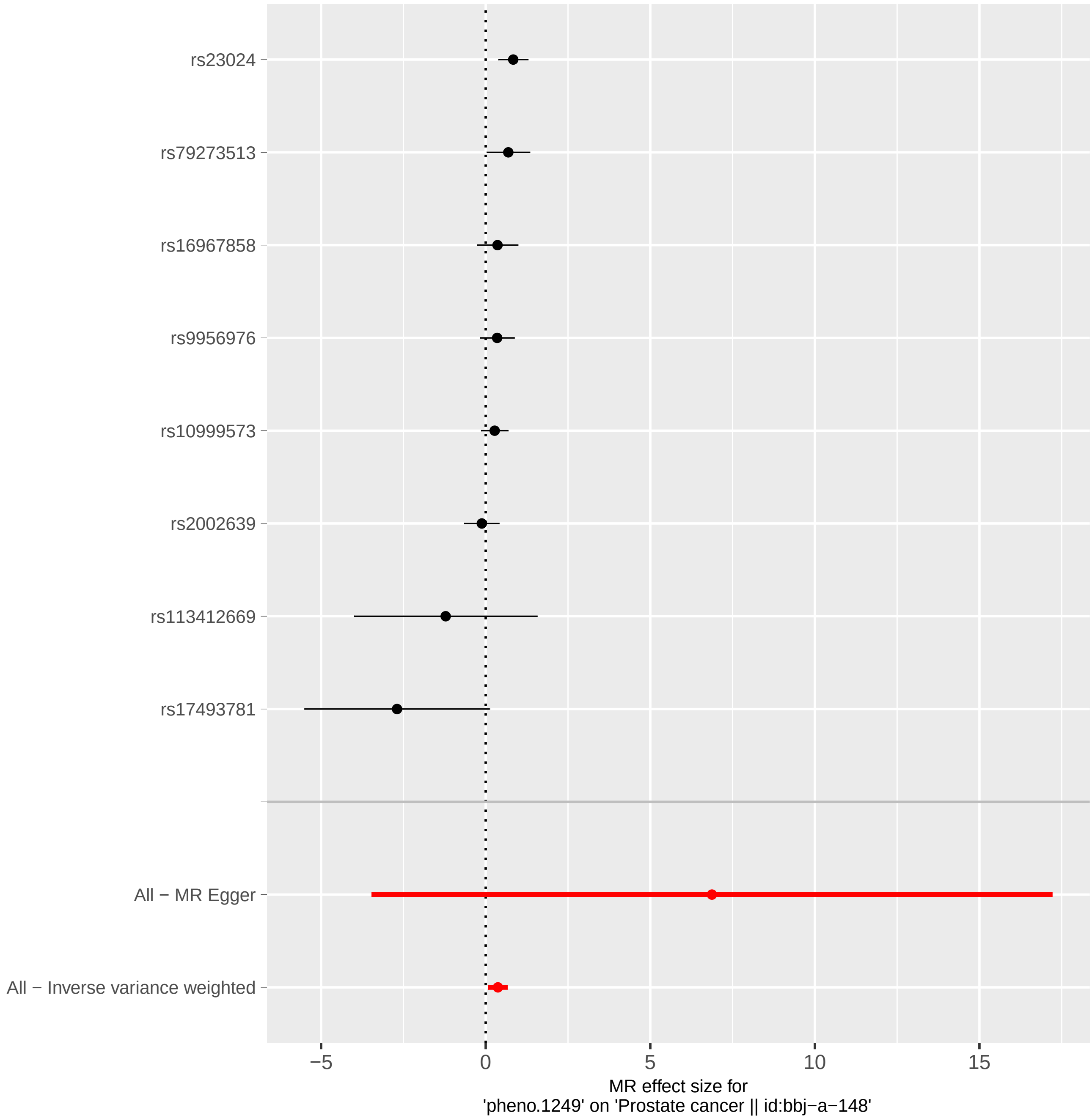
rs23024
rs79273513
rs16967858
rs9956976
rs10999573
rs2002639
rs113412669
rs17493781
All − MR Egger
All − Inverse variance weighted
−5
0
5
10
15
MR effect size for
'pheno.1249' on 'Prostate cancer || id:bbj−a−148'
